# 4-[(4-Bromo­phen­yl)diazen­yl]-2-eth­oxy­aniline

**DOI:** 10.1107/S160053681104877X

**Published:** 2011-11-23

**Authors:** Mohammad Reza Melardi, Jafar Attar Gharamaleki, Soheyla Rezabeyk, Mohammad Kazem Rofouei

**Affiliations:** a Department of Chemistry, Islamic Azad University, Karaj Branch, Karaj, Iran; b Faculty of Chemistry, Tarbiat Moallem University, Tehran, Iran

## Abstract

The title compound, C_14_H_14_BrN_3_O, exhibits a *trans* geometry about the –N=N– double bond. The dihedral angle between the benzene rings is 24.01 (5)°. An intra­molecular N—H⋯O hydrogen bond occurs. In the crystal, inter­molecular N—H⋯N hydrogen bonds between the amine groups lead to the formation of a *C*(8) polymeric chain along [101].

## Related literature

For the synthesis and crystal structures of similar diazenyl compounds, see: de Wit *et al.* (2008 ▶); Yazici *et al.* (2006 ▶). For crystal structure of a chloro analogue of the title compound, see: Rofouei *et al.* (2011 ▶). For graph-set motifs, see: Bernstein *et al.* (1995 ▶).
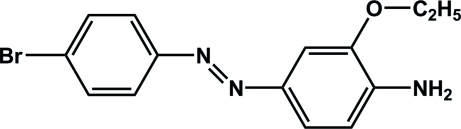


## Experimental

### 

#### Crystal data

C_14_H_14_BrN_3_O
*M* *_r_* = 320.19
Monoclinic, 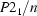

*a* = 13.219 (2) Å
*b* = 8.8289 (17) Å
*c* = 13.506 (2) Å
β = 118.855 (6)°
*V* = 1380.6 (4) Å^3^
*Z* = 4
Mo *K*α radiation
μ = 2.97 mm^−1^
*T* = 200 K
0.40 × 0.20 × 0.10 mm

#### Data collection

Bruker SMART X2S benchtop diffractometer
Absorption correction: multi-scan (*SADABS*; Bruker, 2009 ▶) *T*_min_ = 0.383, *T*_max_ = 0.755
8299 measured reflections
2408 independent reflections
1775 reflections with *I* > 2Σ(*I*)
*R*_int_ = 0.075

#### Refinement

*R*[*F* ^2^ > 2σ(*F* ^2^)] = 0.065
*wR*(*F* ^2^) = 0.198
*S* = 1.00
2408 reflections
178 parameters
2 restraints
H atoms treated by a mixture of independent and constrained refinement
Δρ_max_ = 0.98 e Å^−3^
Δρ_min_ = −1.11 e Å^−3^

### 

Data collection: *SMART X2S* (Bruker, 2009 ▶); cell refinement: *SAINT* (Bruker, 2009 ▶); data reduction: *SAINT*; program(s) used to solve structure: *SHELXS97* (Sheldrick, 2008 ▶); program(s) used to refine structure: *SHELXL97* (Sheldrick, 2008 ▶); molecular graphics: *SHELXTL* (Sheldrick, 2008 ▶); software used to prepare material for publication: *SHELXTL*.

## Supplementary Material

Crystal structure: contains datablock(s) I, global. DOI: 10.1107/S160053681104877X/pv2476sup1.cif

Structure factors: contains datablock(s) I. DOI: 10.1107/S160053681104877X/pv2476Isup2.hkl

Supplementary material file. DOI: 10.1107/S160053681104877X/pv2476Isup3.cml

Additional supplementary materials:  crystallographic information; 3D view; checkCIF report

## Figures and Tables

**Table 1:** Hydrogen-bond geometry (Å, °)

*D*—H⋯*A*	*D*—H	H⋯*A*	*D*⋯*A*	*D*—H⋯*A*
N3—H3*A*⋯N2^i^	0.88 (1)	2.38 (2)	3.228 (6)	163 (5)
N3—H3*B*⋯O1	0.88 (1)	2.28 (5)	2.628 (5)	103 (4)

Symmetry code: (i) 
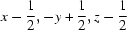
.

**Fig. 1.**
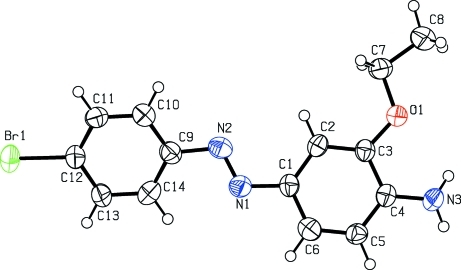
The molecular structure of the title compound, displacement ellipsoids are drawn at 50% probability level.

**Fig. 2.**
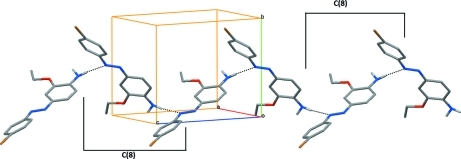
A part of the unit cell showing N3—H3A···N2 hydrogen bonds, to produce polymeric chain of the title molecules along the c axis.

## supplementary crystallographic information

### Comment

We have recenly reported the crystal structure of 4-[(4-chlorophenyl)diazenyl]-3-methoxyaniline (Rofouei *et al.*, 2011), a chloro analogue of the title compound. Diazenyl compounds characterized by having a diazo group (—N═N—) commonly adopt the *trans* configuration in the ground state. In continuation to our work in this field, we now report the crystal structure of the title compound.

The title molecule (Fig. 1) adopts a *trans* configuration about the –N1═N2– double bond and the C1—N1—N2—C9 dihedral angle is 177.3 (4)°. The molecular dimensions in the title compound are similar to the corresponding dimensions reported in other azo compounds (Yazici *et al.*, 2006; de Wit *et al.*, 2008; Rofouei *et al.*, 2011).

In the structure of the title compound, the molecules are linked into chain-like polymers along the *c* axis, with C(8) graph set motif (Bernstein *et al.*, 1995), through N3—H3A···N2^i^ hydrogen bonds with D···A separation of 3.228 (6) Å (Fig. 2 and Tab. 1). The structure is further consolidated by N3—H3A···O1 intramolecular hydrogen bond with D···A separation of 2.628 (5) Å.

### Experimental

To a 100 ml flask in an ice bath, was added (0.349 g, 2 mmol) *p*-boromoaniline and (3.65 g, 0.1 mol) of HCl (d = 1.18 g.ml^-1^). To the obtained solution, was added dropwise a solution of sodium nitrite (0.14 g in 5 ml H_2_O). Then, a diluted solution of *o*-ethoxyaniline (0.244 g, 2 mmol) in methanol (5 ml) was added to the above solution. The *p*H of the solution was adjusted at about 6–7 by adding a solution of 10% of sodium acetate. The solution was stirred for about an hour, giving an orange precipitate. It was then filtered off and dried in vacuum. After dissolving in diethylether and recrystallization, red crystals of the title compound were obtained. *M*.p. 373–376 K.

### Refinement

Hydrogen atoms bonded to carbon were included at geometrically idealized positions and refined in riding mode with distances H—C = 0.95, 0.98 and 0.99 Å for aryl, methyl and methylene type H-atoms, respectively with *U*_iso_(H) set to 1.2(1.5 for methyl)*U*_eq_(C). Hydrogen atoms bonded to N were located from a difference Fourier map and refined with the N—H distances restrained to 0.88 (1) Å and *U*_iso_(H) = 1.2*U*_eq_(N).

### Crystal data

**Table:**

C_14_H_14_BrN_3_O	*F*(000) = 648
*M**_r_* = 320.19	*D*_x_ = 1.540 Mg m^−^^3^
Monoclinic, *P*2_1_/*n*	Mo *K*α radiation, λ = 0.71073 Å
Hall symbol: -P 2yn	Cell parameters from 2935 reflections
*a* = 13.219 (2) Å	θ = 2.9–24.8°
*b* = 8.8289 (17) Å	µ = 2.97 mm^−^^1^
*c* = 13.506 (2) Å	*T* = 200 K
β = 118.855 (6)°	Block, yellow
*V* = 1380.6 (4) Å^3^	0.40 × 0.20 × 0.10 mm
*Z* = 4	

### Data collection

**Table:**

Bruker SMART X2S benchtop diffractometer	2408 independent reflections
Radiation source: fine-focus sealed tube	1775 reflections with *I* > 2Σ(*I*)
graphite	*R*_int_ = 0.075
Detector resolution: 8.33 pixels mm^-1^	θ_max_ = 25.0°, θ_min_ = 1.8°
ω scans	*h* = −15→13
Absorption correction: multi-scan (*SADABS*; Bruker, 2009)	*k* = −10→10
*T*_min_ = 0.383, *T*_max_ = 0.755	*l* = −16→16
8299 measured reflections	

### Refinement

**Table:**

Refinement on *F*^2^	Primary atom site location: structure-invariant direct methods
Least-squares matrix: full	Secondary atom site location: difference Fourier map
*R*[*F*^2^ > 2σ(*F*^2^)] = 0.065	Hydrogen site location: inferred from neighbouring sites
*wR*(*F*^2^) = 0.198	H atoms treated by a mixture of independent and constrained refinement
*S* = 1.00	*w* = 1/[σ^2^(*F*_o_^2^) + (0.1373*P*)^2^] where *P* = (*F*_o_^2^ + 2*F*_c_^2^)/3
2408 reflections	(Δ/σ)_max_ < 0.001
178 parameters	Δρ_max_ = 0.98 e Å^−^^3^
2 restraints	Δρ_min_ = −1.11 e Å^−^^3^

### Special details

**Table:**

Experimental. ^1^H NMR (300 MHz, *d*^6^-DMSO): 1.31 (3*H*, CH_3_), 4.10 (2*H*, OCH_2_), 6.72–7.68 (7*H*, aromatic ring) and 5.87 (2*H*, NH_2_ groups). ^13^C NMR (100 MHz, DMSO): 14.63 (CH_3_), 63.44 (OCH_2_), 101.60–151.34 (C atoms of aromatic rings).
Geometry. All e.s.d.'s (except the e.s.d. in the dihedral angle between two l.s. planes) are estimated using the full covariance matrix. The cell e.s.d.'s are taken into account individually in the estimation of e.s.d.'s in distances, angles and torsion angles; correlations between e.s.d.'s in cell parameters are only used when they are defined by crystal symmetry. An approximate (isotropic) treatment of cell e.s.d.'s is used for estimating e.s.d.'s involving l.s. planes.
Refinement. Refinement of *F*^2^ against ALL reflections. The weighted *R*-factor *wR* and goodness of fit *S* are based on *F*^2^, conventional *R*-factors *R* are based on *F*, with *F* set to zero for negative *F*^2^. The threshold expression of *F*^2^ > σ(*F*^2^) is used only for calculating *R*-factors(gt) *etc*. and is not relevant to the choice of reflections for refinement. *R*-factors based on *F*^2^ are statistically about twice as large as those based on *F*, and *R*- factors based on ALL data will be even larger.

### Fractional atomic coordinates and isotropic or equivalent isotropic displacement parameters (Å^2^)

**Table:**

	*x*	*y*	*z*	*U*_iso_*/*U*_eq_	
Br1	0.62107 (6)	0.84573 (8)	0.08362 (5)	0.0740 (4)	
O1	−0.1368 (3)	0.1305 (4)	−0.0555 (3)	0.0469 (9)	
N1	0.1207 (4)	0.4681 (5)	−0.1255 (3)	0.0441 (10)	
N2	0.1867 (4)	0.4891 (4)	−0.0227 (3)	0.0444 (10)	
N3	−0.2711 (4)	0.1068 (5)	−0.2750 (4)	0.0477 (11)	
H3A	−0.296 (5)	0.071 (6)	−0.343 (2)	0.057*	
H3B	−0.270 (5)	0.039 (5)	−0.227 (3)	0.057*	
C1	0.0220 (4)	0.3782 (5)	−0.1537 (4)	0.0380 (11)	
C2	−0.0054 (4)	0.3036 (5)	−0.0774 (4)	0.0396 (11)	
H2	0.0424	0.3161	0.0018	0.047*	
C3	−0.1012 (4)	0.2134 (5)	−0.1179 (4)	0.0364 (11)	
C4	−0.1750 (4)	0.1953 (5)	−0.2364 (4)	0.0368 (11)	
C5	−0.1446 (4)	0.2697 (5)	−0.3088 (4)	0.0399 (11)	
H5	−0.1917	0.2580	−0.3881	0.048*	
C6	−0.0488 (5)	0.3591 (5)	−0.2688 (4)	0.0438 (13)	
H6	−0.0305	0.4089	−0.3205	0.053*	
C7	−0.0682 (5)	0.1345 (6)	0.0658 (4)	0.0454 (13)	
H7A	−0.0598	0.2399	0.0937	0.054*	
H7B	0.0096	0.0923	0.0900	0.054*	
C8	−0.1313 (5)	0.0403 (7)	0.1108 (4)	0.0601 (15)	
H8A	−0.0876	0.0388	0.1936	0.090*	
H8B	−0.1396	−0.0633	0.0818	0.090*	
H8C	−0.2079	0.0839	0.0864	0.090*	
C9	0.2870 (4)	0.5761 (5)	−0.0032 (4)	0.0429 (12)	
C10	0.3484 (5)	0.6451 (5)	0.0999 (4)	0.0472 (13)	
H10	0.3240	0.6346	0.1552	0.057*	
C11	0.4442 (4)	0.7285 (6)	0.1237 (4)	0.0461 (12)	
H11	0.4841	0.7798	0.1940	0.055*	
C12	0.4832 (4)	0.7385 (6)	0.0462 (4)	0.0479 (13)	
C13	0.4241 (6)	0.6680 (7)	−0.0578 (5)	0.0611 (17)	
H13	0.4510	0.6746	−0.1115	0.073*	
C14	0.3252 (5)	0.5878 (7)	−0.0819 (4)	0.0566 (15)	
H14	0.2829	0.5401	−0.1534	0.068*	

### Atomic displacement parameters (Å^2^)

**Table:**

	*U*^11^	*U*^22^	*U*^33^	*U*^12^	*U*^13^	*U*^23^
Br1	0.0634 (5)	0.0968 (6)	0.0643 (5)	−0.0445 (4)	0.0328 (4)	−0.0213 (3)
O1	0.043 (2)	0.060 (2)	0.0371 (17)	−0.0123 (17)	0.0188 (17)	−0.0022 (15)
N1	0.046 (3)	0.043 (2)	0.040 (2)	−0.0016 (19)	0.018 (2)	0.0031 (17)
N2	0.049 (3)	0.043 (2)	0.043 (2)	0.0051 (19)	0.024 (2)	0.0064 (18)
N3	0.045 (3)	0.055 (3)	0.041 (2)	−0.014 (2)	0.019 (2)	−0.0120 (19)
C1	0.034 (3)	0.037 (2)	0.044 (3)	−0.002 (2)	0.020 (2)	−0.0029 (19)
C2	0.031 (3)	0.043 (2)	0.039 (2)	0.000 (2)	0.012 (2)	−0.001 (2)
C3	0.031 (3)	0.038 (2)	0.042 (3)	0.001 (2)	0.019 (2)	−0.002 (2)
C4	0.032 (3)	0.035 (2)	0.043 (3)	0.001 (2)	0.018 (2)	−0.0042 (19)
C5	0.041 (3)	0.043 (3)	0.037 (3)	−0.001 (2)	0.020 (2)	−0.005 (2)
C6	0.055 (3)	0.036 (2)	0.049 (3)	0.000 (2)	0.031 (3)	−0.002 (2)
C7	0.049 (3)	0.049 (3)	0.037 (3)	−0.001 (2)	0.019 (2)	−0.001 (2)
C8	0.068 (4)	0.060 (3)	0.050 (3)	−0.010 (3)	0.028 (3)	0.005 (3)
C9	0.044 (3)	0.043 (3)	0.045 (3)	0.000 (2)	0.023 (2)	0.005 (2)
C10	0.050 (3)	0.049 (3)	0.046 (3)	−0.004 (2)	0.025 (3)	0.002 (2)
C11	0.049 (3)	0.047 (3)	0.036 (3)	−0.004 (2)	0.017 (2)	0.000 (2)
C12	0.040 (3)	0.054 (3)	0.046 (3)	−0.012 (2)	0.018 (2)	0.001 (2)
C13	0.062 (4)	0.081 (4)	0.050 (3)	−0.034 (3)	0.035 (3)	−0.016 (3)
C14	0.050 (3)	0.068 (4)	0.043 (3)	−0.014 (3)	0.015 (3)	−0.010 (3)

### Geometric parameters (Å, °)

**Table:**

Br1—C12	1.893 (5)	C6—H6	0.9500
O1—C3	1.361 (6)	C7—C8	1.498 (8)
O1—C7	1.439 (6)	C7—H7A	0.9900
N1—N2	1.246 (5)	C7—H7B	0.9900
N1—C1	1.412 (6)	C8—H8A	0.9800
N2—C9	1.442 (7)	C8—H8B	0.9800
N3—C4	1.363 (7)	C8—H8C	0.9800
N3—H3A	0.876 (10)	C9—C10	1.371 (7)
N3—H3B	0.879 (10)	C9—C14	1.383 (8)
C1—C6	1.384 (7)	C10—C11	1.363 (7)
C1—C2	1.410 (7)	C10—H10	0.9500
C2—C3	1.367 (7)	C11—C12	1.375 (7)
C2—H2	0.9500	C11—H11	0.9500
C3—C4	1.424 (7)	C12—C13	1.383 (7)
C4—C5	1.388 (7)	C13—C14	1.380 (9)
C5—C6	1.363 (7)	C13—H13	0.9500
C5—H5	0.9500	C14—H14	0.9500
			
C3—O1—C7	118.4 (4)	O1—C7—H7B	110.5
N2—N1—C1	116.3 (4)	C8—C7—H7B	110.5
N1—N2—C9	111.8 (4)	H7A—C7—H7B	108.7
C4—N3—H3A	114 (4)	C7—C8—H8A	109.5
C4—N3—H3B	116 (4)	C7—C8—H8B	109.5
H3A—N3—H3B	113 (5)	H8A—C8—H8B	109.5
C6—C1—C2	119.4 (4)	C7—C8—H8C	109.5
C6—C1—N1	114.1 (4)	H8A—C8—H8C	109.5
C2—C1—N1	126.5 (4)	H8B—C8—H8C	109.5
C3—C2—C1	119.6 (4)	C10—C9—C14	119.4 (5)
C3—C2—H2	120.2	C10—C9—N2	117.6 (5)
C1—C2—H2	120.2	C14—C9—N2	123.0 (5)
O1—C3—C2	126.5 (4)	C11—C10—C9	120.4 (5)
O1—C3—C4	112.5 (4)	C11—C10—H10	119.8
C2—C3—C4	121.0 (4)	C9—C10—H10	119.8
N3—C4—C5	122.3 (4)	C10—C11—C12	120.2 (5)
N3—C4—C3	120.0 (4)	C10—C11—H11	119.9
C5—C4—C3	117.7 (4)	C12—C11—H11	119.9
C6—C5—C4	121.6 (4)	C11—C12—C13	120.6 (5)
C6—C5—H5	119.2	C11—C12—Br1	119.7 (4)
C4—C5—H5	119.2	C13—C12—Br1	119.7 (4)
C5—C6—C1	120.8 (5)	C14—C13—C12	118.4 (5)
C5—C6—H6	119.6	C14—C13—H13	120.8
C1—C6—H6	119.6	C12—C13—H13	120.8
O1—C7—C8	106.3 (4)	C13—C14—C9	120.9 (5)
O1—C7—H7A	110.5	C13—C14—H14	119.6
C8—C7—H7A	110.5	C9—C14—H14	119.6
			
C1—N1—N2—C9	177.3 (4)	C2—C1—C6—C5	0.2 (7)
N2—N1—C1—C6	179.6 (4)	N1—C1—C6—C5	177.1 (4)
N2—N1—C1—C2	−3.8 (7)	C3—O1—C7—C8	177.1 (5)
C6—C1—C2—C3	0.2 (7)	N1—N2—C9—C10	161.2 (5)
N1—C1—C2—C3	−176.3 (5)	N1—N2—C9—C14	−21.2 (7)
C7—O1—C3—C2	−0.6 (7)	C14—C9—C10—C11	2.1 (8)
C7—O1—C3—C4	178.2 (4)	N2—C9—C10—C11	179.8 (4)
C1—C2—C3—O1	177.6 (5)	C9—C10—C11—C12	−3.0 (8)
C1—C2—C3—C4	−1.0 (7)	C10—C11—C12—C13	1.9 (9)
O1—C3—C4—N3	2.2 (6)	C10—C11—C12—Br1	−176.6 (4)
C2—C3—C4—N3	−178.9 (5)	C11—C12—C13—C14	0.1 (10)
O1—C3—C4—C5	−177.4 (4)	Br1—C12—C13—C14	178.6 (5)
C2—C3—C4—C5	1.5 (7)	C12—C13—C14—C9	−1.0 (10)
N3—C4—C5—C6	179.3 (5)	C10—C9—C14—C13	−0.1 (9)
C3—C4—C5—C6	−1.1 (7)	N2—C9—C14—C13	−177.6 (6)
C4—C5—C6—C1	0.3 (8)		

### Hydrogen-bond geometry (Å, °)

**Table:**

*D*—H···*A*	*D*—H	H···*A*	*D*···*A*	*D*—H···*A*
N3—H3A···N2^i^	0.88 (1)	2.38 (2)	3.228 (6)	163 (5)
N3—H3B···O1	0.88 (1)	2.28 (5)	2.628 (5)	103 (4)

Symmetry codes: (i) *x*−1/2, −*y*+1/2, *z*−1/2.
